# Chronic Constipation Unmasked: A Comprehensive Case Study of Colonic Inertia and Its Future Implications

**DOI:** 10.7759/cureus.60031

**Published:** 2024-05-10

**Authors:** Enaiya J Awan, Kyle W Plunk, Arielle N Washington, Matias G Buedo, Jayson Messieh, Alexandra B Contardi, Rachel MpanuMpanu, Armaan M Sobhan, Eliyahu Shemesh, Andrew M O'Neill, Feras Othman

**Affiliations:** 1 Medical School, St. George's University School of Medicine, Great River, USA; 2 Medical School, Ross University School of Medicine, Miami, USA; 3 Colorectal Surgery, Delray Medical Center, Delray Beach, USA; 4 Minimally Invasive Surgery, Delray Medical Center, Delray Beach, USA; 5 General Surgery, University of Miami, Coral Gables, USA

**Keywords:** chronic abdominal pain, gastrointestinal disease, colonic motility, subtotal colectomy with ileostomy, constipation, colonic inertia

## Abstract

Colonic inertia is a gastrointestinal disorder characterized by a significant delay in colon transit, resulting in chronic constipation that impedes an undisclosed percentage of individuals in the United States. This article aims to delve into the intricate mechanisms underlying the hindered transit observed in colonic inertia, focusing on multifactorial etiology and treatment. By gaining a better understanding of the pathophysiology of colonic inertia, we can improve the quality of life for individuals affected by this condition. Our study employs a comprehensive approach, combining clinical observation during pancolectomy, histopathological analyses performed by pathologists, and detailed investigation to unravel the complex interplay of factors affecting colonic motility.

## Introduction

Chronic constipation affects 12-19% of North American gastrointestinal outpatient cases, with an 8.2% higher prevalence in females, individuals over the age of 60 with a sedentary lifestyle and low-fiber diet, and low-income populations [1]. Colonic inertia is a pathological condition defined by slow or ineffective colon movement, leading to chronic constipation. The etiology of colonic inertia has been theorized to be related to a decrease in spontaneous high-amplitude propagating sequences, a decrease in the number and frequency of cyclic motor patterns after high-calorie meals, or a decline in interstitial cells of Cajal [2]. Any disruption in the signaling between these components or various factors such as neuromuscular dysfunction, abnormalities in the enteric nervous system, hormonal imbalances, lifestyle factors, idiopathic causes, and certain pharmacological therapies such as opioids can interfere with normal bowel movements [1]. 

The enteric nervous system also plays a critical role in coordinating secretion, absorption, and motility within the gastrointestinal system. Abnormalities in the enteric nervous system such as neuropathies or myopathies can contribute to a slow transit constipation due to uncoordinated signals. 

Hormonal imbalances are also indicated as a potential cause of colonic inertia. Low thyroid conditions such as hypothyroidism reduce peristaltic movement leading to bowel hypomotility leading to chronic constipation [3]. Additionally, lifestyle factors such as low fiber, dehydration, and inadequate physical activity can also contribute to ineffective colon movement. 

Clinically, colonic inertia can present with symptoms of infrequent bowel movements, abdominal discomfort, bloating, and incomplete evacuation. Once the diagnosis is confirmed by the sitz marker study and radiological imaging, first-line treatment is with laxatives such as polyethylene glycol, linaclotide, or lubiprostone [4]. 

Although further research is required, acid-suppressive drugs such as proton pump inhibitors have been linked as additions to the treatment of colonic inertia. Proton pump inhibitors can increase the amount of available physiologically active substances and enhance the metabolic environment of the gut by decreasing pathogenic microbiota that accumulated due to delayed colonic transit, thereby enhancing the absorption of constipation treatment [5]. 

Finally, patients with persistent symptoms who are resistant to medications along with the exclusion of psychiatric causes, a proven delay in colonic transit, and the absence of pelvic floor dysfunction can benefit from a pancolectomy [6].

## Case presentation

**Figure 1:**
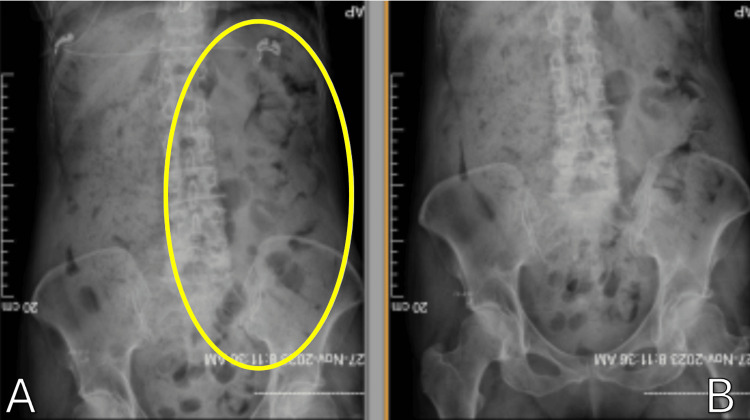
(A-B) Abdominal X-ray revealing heavy stool burden

**Figure 2:**
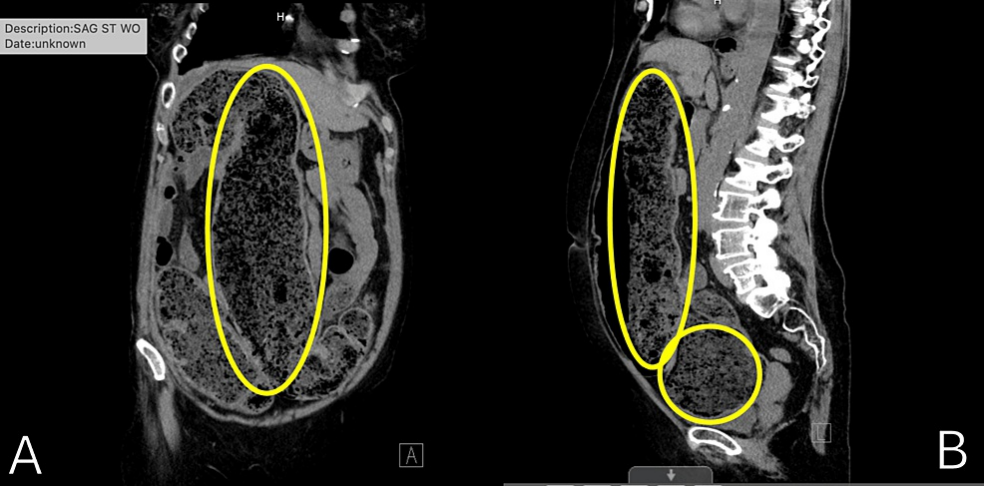
(A) Coronal CT of the abdomen showing colonic dilatation of the ascending and transverse colon. (B) Sagittal CT of the abdomen revealing colonic dilation of the transverse colon

This is a case of a 75-year-old female with a long-standing medical history of constipation refractory to years of stool softeners and laxatives. The patient's medical history includes controlled hypothyroidism treated with hormone replacement therapy as well as osteopenia being managed by her primary care provider. The patient was referred to a dietician for correction or substitutions in her diet without resolution of her symptoms. Despite various approaches, her primary care provider ultimately advised a colorectal consultation for refractory constipation. She underwent various investigations as a workup for her condition including an abdominal X-ray (Figure 1) which revealed a heavy stool burden and a CT scan that showed a markedly dilated colon with features of fecal impaction (Figure 2). The diagnostic evaluation involved a review of clinical history, physical examination, as well as laboratory markers revealing mild hypokalemia, hypocalcemia, and anemia.

**Figure 3:**
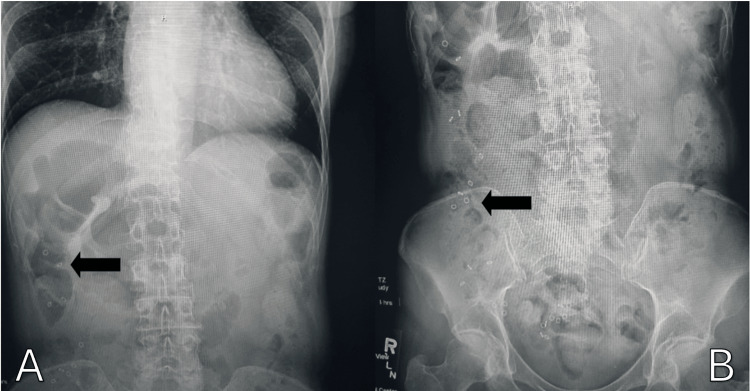
(A) AP X-ray with sitz marker. (B) AP X-ray with sitz marker: sitz marker study with marker residing mainly in the ascending colon after 48 hours AP: anteroposterior

**Figure 4:**
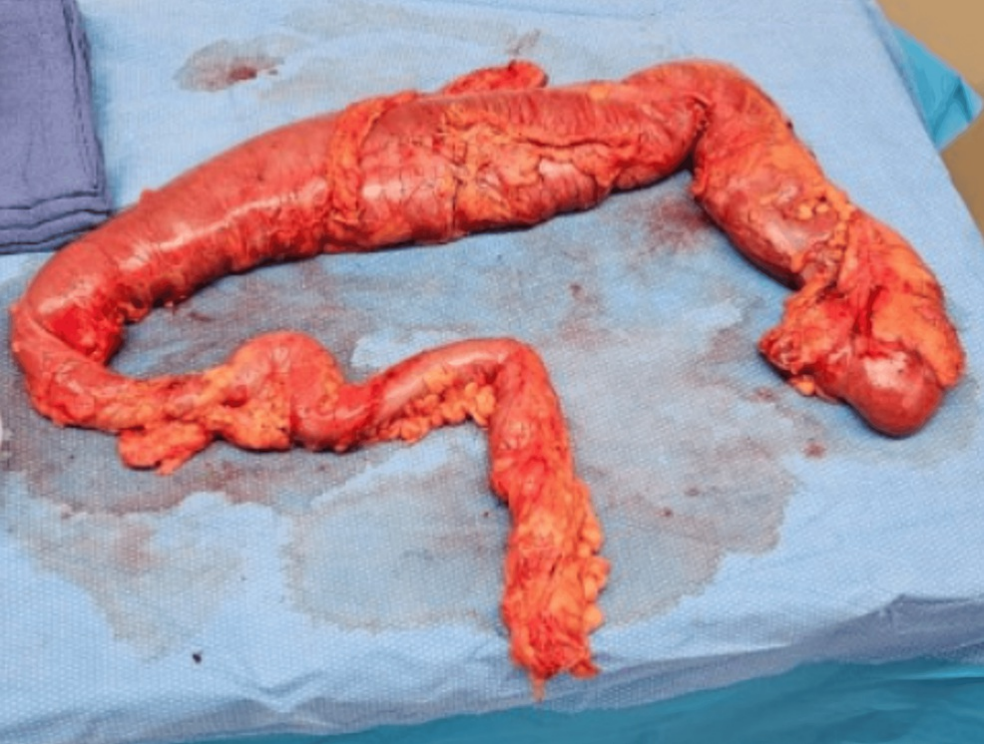
Gross specimen of subtotal colectomy

Colonoscopy was suboptimal, identifying pan-colonic dilatation with healthy mucosa without suspicious masses or obstructive lesions. Colonic inertia and rectal intussusception were major aspects of the differential diagnosis, so she underwent a sitz marker study (Figure 3) which showed delayed colonic transit time and the presence of the sitz markers in the hepatic flexure after four days of ingestion. Her condition was not appropriately responding to conservative treatment, so the decision was made to undergo a subtotal colectomy (Figure 4). The intraoperative finding was positive for a remarkably dilated colon. During the two-week follow-up clinic visit, the patient reported mild but improving diarrhea with increased regularity and formed stools. By the fourth week postoperative visit, her stool had become regular with normal daily bowel movements.

## Discussion

As part of the colonic inertia workup, differential diagnoses include Hirschsprung's disease, chronic opioid use, degenerative joint disease, and rectal intussusception. Hirschsprung's disease is distinguished by histopathological findings, such as absent ganglion cells, and may involve genetic abnormalities like trisomy 21 [7].

Hypothyroidism can also impair peristalsis, leading to constipation [8]. Lifestyle factors such as low fiber intake, dehydration, and physical inactivity contribute to colon motility issues. Opioids, binding to gut receptors, decrease motility and stool transit time, causing constipation and potentially fecal impaction [9]. Degenerative joint disease, inducing pain and often treated with opioids, often promotes sedentary behavior, exacerbating colonic inertia in regard to lack of activity. Rectal intussusception presents as distinct symptoms including constipation, bleeding, and mucosal discharge [10]. In contrast, colonic inertia involves neuromuscular dysfunction, contrasting with structural lesions in rectal intussusception. Differential diagnosis includes a sitz marker study to assess transit time to differentiate between the two [11].

Treatment options for colonic inertia vary depending on symptom severity and individual circumstances. General approaches may include laxatives, prokinetic agents, dietary changes, hydration, biofeedback therapy, physical activity, behavioral modifications, and surgery, such as colectomy in severe refractory cases. Consultation with a healthcare professional is essential to tailor the treatment plan to the patient's needs and symptoms [2]. A multidisciplinary approach where the more dangerous etiology is ruled out as well as close monitoring by the patient's primary care provider is important to the successful management of symptoms [12].

The patient's history of chronic constipation led to a subtotal colectomy and ileostomy due to refractory symptoms, including marked colonic dilatation and fecal impaction. Postoperative histopathology showed no abnormalities, contrasting with the typical histological findings of massive submucosal ganglia in colonic inertia [13-14]. Her initial presentation included diffuse abdominal pain, constipation, nausea, and vomiting, with imaging revealing massive colonic distention without perforation. Notably, the patient lacked known predisposing factors for colonic inertia apart from age and gender, which are associated with chronic constipation.

Conservative measures, including bisacodyl and polyethylene glycol, proved ineffective, eventually leading to subtotal colectomy due to the severity of the patient's presentation with marked stool impaction and abdominal distention (Figure 4).

## Conclusions

Colonic inertia is an area of ongoing research, as the direct pathophysiology of its development is not fully understood, and a variety of factors may influence its development. As it may be described by the neurohormonal disinhibition between the enteric nervous system, or simply the sequela of a patient's lifestyle and age-related changes, its dysfunctional nature should be managed to prioritize the patient's quality of life and improve symptoms through early identification with behavioral and pharmacological treatment.
